# Hexagonal Boron Nitride on III–V Compounds: A Review of the Synthesis and Applications

**DOI:** 10.3390/ma15134396

**Published:** 2022-06-22

**Authors:** Yufei Yang, Yi Peng, Muhammad Farooq Saleem, Ziqian Chen, Wenhong Sun

**Affiliations:** 1 Research Center for Optoelectronic Materials and Devices, Guangxi Key Laboratory for the Relativistic Astrophysics, School of Physical Science and Technology, Guangxi University, Nanning 530004, China; 17729828140@163.com (Y.Y.); 1907401031@st.gxu.edu.cn (Y.P.); 2 MOE Key Laboratory of New Processing Technology for Nonferrous Metals and the Guangxi Key of Processing for Non-Ferrous Metals and Featured Materials, Nanning 530004, China; 3 GBA Branch of Aerospace Information Research Institute, Chinese Academy of Sciences, Guangzhou 510700, China

**Keywords:** hexagonal boron nitride, wide-band-gap semiconductor, physical vapor deposition, chemical vapor deposition, co-segregation method

## Abstract

Since the successful separation of graphene from its bulk counterpart, two-dimensional (2D) layered materials have become the focus of research for their exceptional properties. The layered hexagonal boron nitride (h-BN), for instance, offers good lubricity, electrical insulation, corrosion resistance, and chemical stability. In recent years, the wide-band-gap layered h-BN has been recognized for its broad application prospects in neutron detection and quantum information processing. In addition, it has become very important in the field of 2D crystals and van der Waals heterostructures due to its versatility as a substrate, encapsulation layer, and a tunneling barrier layer for various device applications. However, due to the poor adhesion between h-BN and substrate and its high preparation temperature, it is very difficult to prepare large-area and denseh-BN films. Therefore, the controllable synthesis of h-BN films has been the focus of research in recent years. In this paper, the preparation methods and applications of h-BN films on III–V compounds are systematically summarized, and the prospects are discussed.

## 1. Introduction

Since graphene was separated from graphite 18 years ago, research on 2D materials has entered a new era [1,2,3,4]. Since then, the 2D structure of h-BN and similar layered materials has been the focus of research [5,6,7,8,9]. The development of high-quality van der Waals heterostructures and new photoelectric applications highlight the excellent performance of 2D h-BN. h-BN is a 2D electrical insulator. The insulating behavior of h-BN comes from its large band gap, which is up to 5.8 eV [8].

In 1984, Wigging et al. [10] deposited BN films with a thickness of 1 µm on sapphire and silicon substrates by using magnetron sputtering. In 1990, Mieno et al. [11] used sintered h-BN target to deposit BN on a silicon substrate using radio frequency magnetron sputtering technology and found that BN was a mixture of c-BN and h-BN. In 2017, Jitsuo et al. [12] deposited an Fe catalytic layer on an AlN substrate. They used high-purity B as the target material and Ar/H_2_ as the working gas, and a high-quality h-BN thin film was deposited on the Fe/AlN heterogeneous interface using pulse magnetron sputtering technology. In the early stage, h-BN films were usually prepared with physical deposition methods, but it was usually difficult to prepare large-area, high-quality h-BN films. In 2010, Li Song et al. [13] used the atmospheric pressure chemical vapor deposition (APCVD) method to prepare large-area h-BN thin films on copper foil using aminoborane as precursor and Ar/H_2_ as a carrier gas. In 2011, R. Dahl et al. [14] used metal–organic chemical vapor deposition (MOCVD) method technology to prepare micron-scale high-quality h-BN films on sapphire substrates using triethyl boron and ammonia as precursors. Using chemical vapor deposition (CVD) method technology, large-area, single-crystal h-BN and many other layered materials have been synthesized, making it the first choice for high-quality epitaxial growth.

**Figure 1:**
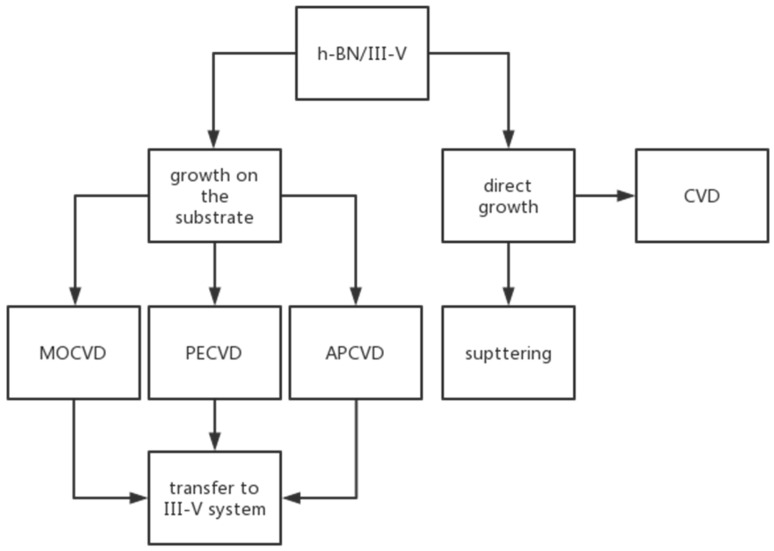
h-BN/III–V preparation methods.

In earlier studies, researchers found it difficult to directly grow high-quality graphene on substrates such as sapphire. The Figure 1 shows the h-BN/III–V preparation methods. Due to the structural similarity between h-BN and graphene, h-BN is known as a more suitable substrate for graphene growth. h-BN has strong atomic affinity, a smooth surface, and good thermal stability, and the preparation of graphene devices with boron nitride as the substrate can improve electron mobility. Dean et al. [15] prepared graphene on h-BN and SiO_2_ substrates, and the results showed that the electron mobility of graphene on h-BN is 10 times higher than that on SiO_2_. In recent years, UV-LEDs with GaN/AlGaN as the main material have been widely used in the fields of disinfection and sterilization, but GaN/AlGaN devices suffer low luminous efficiency and low surface electronic activity. Due to the improved performances of graphene/h-BN devices, h-BN based heterostructures such as h-BN/GaN(AlN) have become the focus of research. This paper systematically summarizes the structure, growth, and applications of h-BN and its heterostructures.

### 1.1. Crystal Structure

BN is a III–V compound with an equal number of B and N atoms. BN is mainly obtained with chemical and physical synthesis methods, while a few natural BN-based materials are also found in nature [16]. BN mainly crystallizes in two stable phases: cubic (c-BN), hexagonal (h-BN), and two metastable phases: wurtzite (w-BN) and rhombus (r-BN) (Table 1).

**Figure 2:**
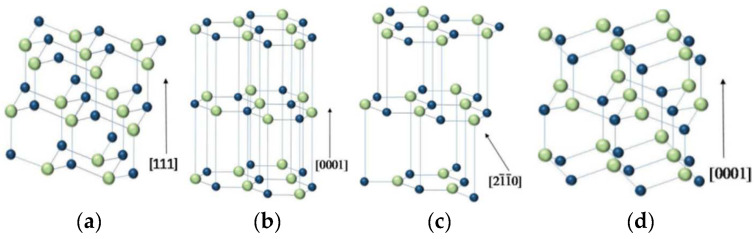
Schematic diagram of the crystal structure of various BN phases [17].; (**a**) c-BN; (**b**) h-BN; (**c**) r-BN; (**d**) w-BN.

**Figure 3:**
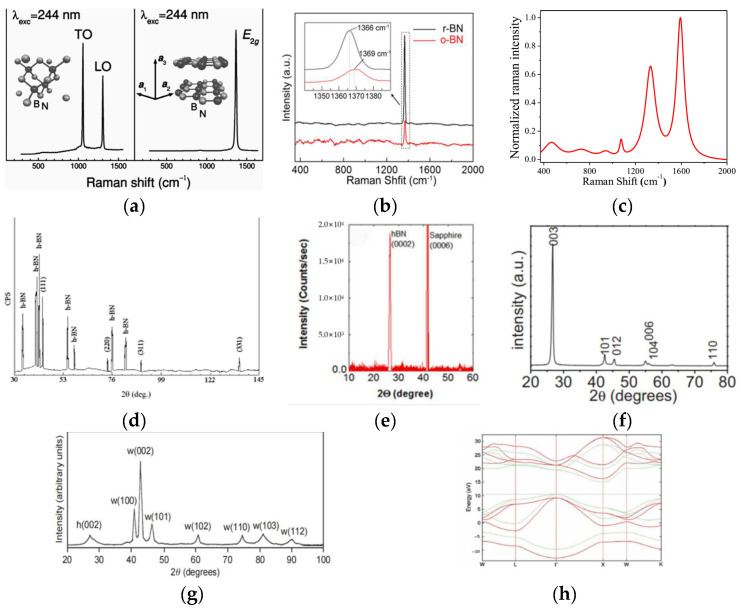
Raman for c-BN (**a**) left [18], h-BN (**a**) right [18], r-BN (**b**) [19], w-BN (**c**) [20]. XRD for c-BN (**d**) [21], h-BN (**e**) [14], r-BN (**f**) [22], w-BN (**g**) [23]. (**h**) Band structure of h-BN along the high symmetry directions [24].

As shown in Figure 2a, c-BN has a regular tetrahedral structure with lattice constant a = 0.3615 nm. The Figure 3a left and Figure 3d show the Raman and XRD for the c-BN. Each B (N) atom is connected to three N (B) atoms. The bond energy of this covalent bond even exceeds that of the c–c bond in diamond with a similar structure. Therefore, the thermal stability of c-BN is higher than that of diamond. The atomic layer arrangement of c-BN is ABCABC, and this structure can be regarded as a compound lattice formed by two face-centered cubic structures staggered by one quarter of the diagonal distance between each other. Each atom is at the vertex of the regular tetrahedron and forms four covalent bonds.

As shown in Figure 2b, h-BN has a hexagonal, mesh-like layered structure, and its stacking order is AAAA. In the horizontal direction, B and N atoms are in the same plane, while in the vertical direction, B atoms are on top of the N atoms, which is called Bernal stacking [25]. The Figure 3a right and Figure 3e show the Raman and XRD for the h-BN. The Figure 3h shows band structure of h-BN. h-BN powder is white at room temperature. h-BN is also known as “white stone ink” because of its similar structure to graphite (Table 2). The bond between B and N atoms in h-BN is formed by sp^2^ hybridization, and the bond between layers is formed by the van der Waals force. Although h-BN and graphite have similar structures, they are quite different in electrical properties. The reason for this difference is that the layered structure of h-BN produces half hexagonal mesh dislocations every other layer, which makes it impossible to conduct electricity due to the lack of free electrons [26,27].

r-BN is a tripartite crystal system and belongs to an orthogonal center structure. As shown in Figure 2c, its structure is similar to h-BN. The Figure 3b,f show the Raman and XRD for the r-BN. A weaker van der Waals force between layers makes it easier to slide between layers. r-BN is the metastable phase of h-BN, which is usually accompanied by the formation of r-BN during the preparation of h-BN. It is very difficult to separate these two phases by physical methods; r-BN has poor stability at high temperatures and converts to h-BN.

w-BN is a hexagonal crystal system, bonding between B and N atoms in the form of sp^3^ hybridization and stacking in the form of ABAB. The special feature of its atomic structure lies in the fact that the same atoms are on the same plane (one layer of B atoms and one layer of N atoms), as shown in Figure 2d. The Figure 3c,g show the Raman and XRD for the h-BN.The lattice constant of w-BN is a = 0.255 nm and c = 0.422 nm. As a metastable phase of c-BN, w-BN is very similar to c-BN in physical and chemical properties. w-BN has a synthetic structure with excellent electrical insulation and high industrial value [29,30].

### 1.2. The Interface Properties of Various h-BN Heterostructures

**Figure 4:**
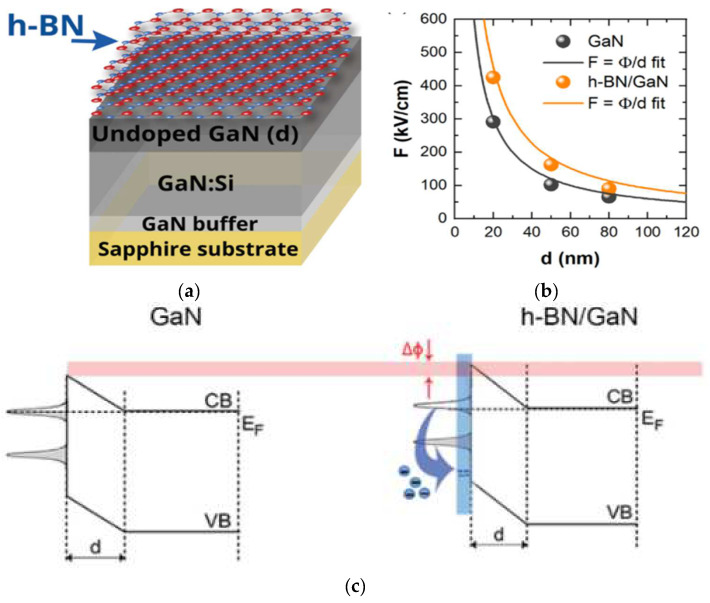
(**a**) h-BN/GaN structure. (**b**) Determination of surface barrier height for GaN and h-BN/GaN structures together with the fitting curves. (**c**) The lowering of Fermi level on the h-BN/GaN interface caused by h-BN [32].

Although III–V compounds are widely used in light-emitting diodes, photodetectors, and transistors, leakage current and thermal conductivity still affect device performance. Table 3 compares the data of h-BN, GaN and AlN.The Fermi level at the interface of the heterogeneous structure is the key to successful device operation. The position of the Fermi level on the surface is determined by the impurities and defects introduced during the growth process [31], thus affecting the performance of devices. Ewelina Zdanowicz et al. [32] used the contactless electroreflectance (CER) method to study the electrical properties of the h-BN/GaN interface. They deposited a layer of h-BN thin film on GaN using the metal–organic chemical vapor deposition (MOCVD) technique (Figure 4a) and studied the properties of its interface effect. The experimental results showed that h-BN lowers the Fermi level position and increases the surface barrier (Figure 4b). A charge transfer from GaN to the h-BN acceptor state was observed. The results indicated that h-BN can reduce dark current, change the excited state of electrons on the GaN surface (Figure 4c), support electron blocking, and provide effective hole injection into the active region to improve device performance.

**Figure 5:**
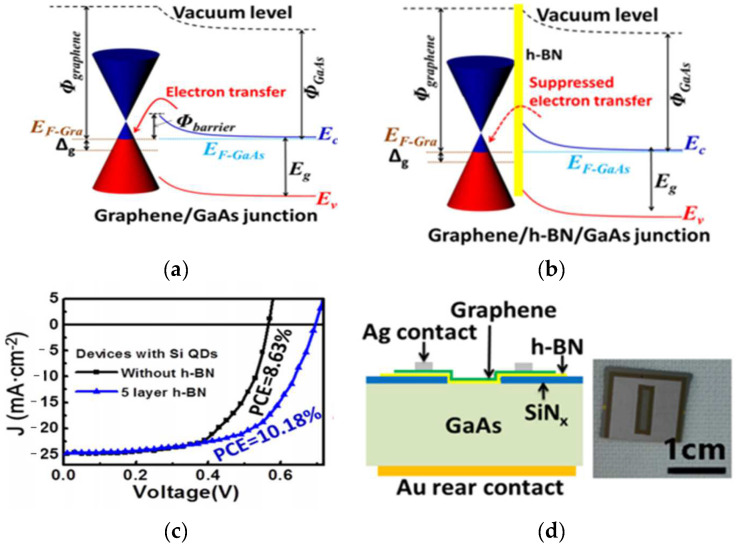
(**a**) Band structure of graphene/GaAs heterojunction. (**b**) Band structure of graphene/h-BN/GaAs heterojunction. (**c**) J–V curves of solar cells based on graphene/GaAs and graphene/h-BN/GaAs heterostructure with Si QDs introduced using photo-induced doping. (**d**) Left: schematic structure of the graphene/h-BN/GaAs sandwich device; right: digital photograph of one typical device [46].

With its excellent electronic, optical, and mechanical properties, graphene has become a popular material for manufacturing a new generation of optoelectronic devices [1,34,35,36], including high intrinsic carrier mobility [37], a wide absorption spectrum [38], high Young’s modulus [39], and excellent thermal conductivity [40]. Graphene is an excellent photocurrent converter with an internal quantum efficiency approaching 100% [41], but the absorption rate of single-atom layer graphene is too low to obtain high-performance optoelectronic devices [38]. To obtain high-performance graphene devices [42,43,44], the heterostructures of graphene with bulk semiconductors have become feasible in many experimental schemes [45]. On the one hand, the height of the potential barrier between graphene and the semiconductor interface greatly affects the volt–current performance. On the other hand, charge transfers between graphene and semiconductors affect device performance. Xiaoqiang Li et al. [46] prepared Graphene/h-BN/GaAs sandwich diode (Figure 5d) and compared its performance to a diode without h-BN. The open-circuit voltage (Voc) of the graphene/GaAs device was found to be 0.56 V, which increased to 0.66 V after adding h-BN, and the power conversion efficiency (PCE) also increased from 6.51% to 7.10%. In addition, the PCE of graphene/GaAs structure can reach 8.63%, and the PCE of graphene/h-BN/GaAs structure can reach 10.18% after photoinduced doping (Figure 5c), which greatly improves the photoelectric detection performance of the device.

## 2. The Application of h-BN

The earlier h-BN heterostructures are mainly electronic devices composed of graphene. In recent years, it has been found that the heterogeneous combination of h-BN and GaN(AlN) can effectively improve the performance of GaN(AlN)-based optoelectronic devices. For transistor structures, h-BN acts as a passivation layer and gate dielectric, neutralizing surface traps and reducing leakage current. In photodetectors, it reduces the dark current.

### 2.1. Heat Dissipation of h-BN

**Figure 6:**
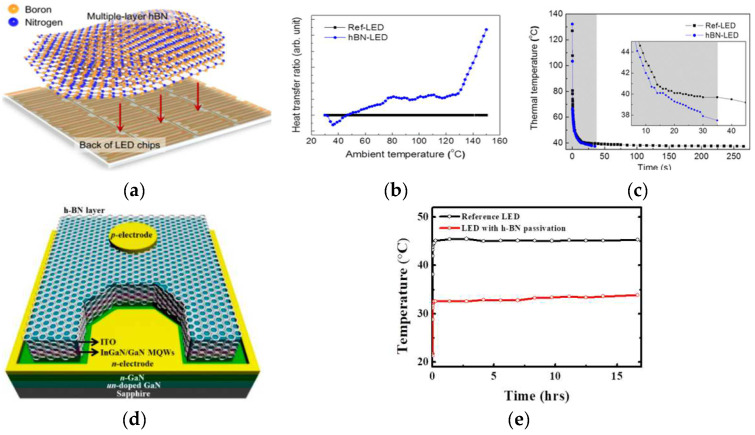
(**a**) Schematic representation of the transfer process of multiple-layer h-BN onto the back of an LED wafer. (**b**) HTR values of the LEDs as a function of ambient temperature, obtained from DSC experiments. (**c**) Tmax as a function of time after stopping the injection of current into the LED chips [47]. (**d**) Schematic diagram of fabricated InGaN/GaN-based light-emitting diode (LED) with hexagonal boron nitride (h-BN) film as a passivation layer. (**e**) Operation temperatures on both devices as a function of operation time at 100 mA [49].

GaN-based LEDs fabricated on sapphire usually have low heat dissipation efficiency, which is caused by the poor heat dissipation capacity of the substrate itself. h-BN has high thermal conductivity (TC) and the potential to be an excellent heat-conducting medium. Ilgyu Choi et al. [47] made a comparison between the heat dissipation of h-BN-LED (Figure 4a) and an ordinary LED through experiments, and the results showed that the attachment of h-BN improves the heat dissipation capacity of the device by about seven times (Figure 6b,c). Generally, the main heat dissipation mode of GaN-based LEDs is air convection in the vertical direction, and the heat dissipation is insufficient due to the poor heat dissipation capacity of the epitaxial layer and substrates. As 2D h-BN has a high TC, it increases the horizontal heat dissipation ability and enhances the durability of the device after attaching h-BN. AlGaN/GaN high electron mobility transistors (HEMTs) are used in high frequency and high power applications due to their inherent III–V characteristics, such as high electron saturation rates and high critical electric fields [48]. SiN is usually used for GaN surface passivation to suppress device current collapse. GaN has a poor heat dissipation ability, particularly for high performance devices. Gun-Hee Lee et al. [49] Prepared InGaN/GaN light-emitting diodes (LEDs) with h-BN as a passivation layer (Figure 6d). The h-BN layer minimized the leakage current characteristics and operating temperature by acting as a passivation and heat dispersion layer. With a reduced working temperature of 33 from 45 °C, the LED lifetime was extended by 2.5 times following h-BN passivation (Figure 6e).

### 2.2. The Passivation of h-BN

**Figure 7:**
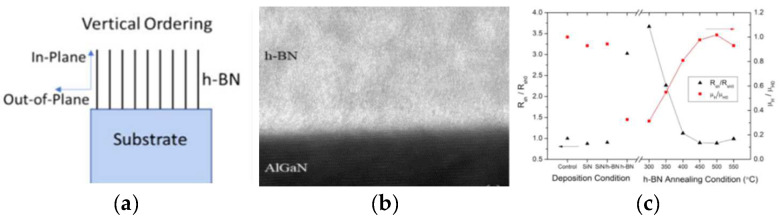
(**a**) Schematic diagram of vertically ordered h-BN on the substrate. (**b**) Cross-sectional HRTEM images of h-BN on AlGaN/GaN HS. (**c**) Normalized sheet resistance (Rsh) and 2DEG mobility (μH) of AlGaN/GaN HSs versus the different deposition conditions (control, after SiN, after h-BN/SiN, after h-BN deposition, and after postdeposition annealing temperatures). For postdeposition annealing, each of the samples was subjected to annealing at their respective temperatures for 300 s [50,51].

The h-BN structure is similar to graphene with relatively high plane thermal conductivity and chemical stability, which makes it suitable for device surface passivation. Matthew Whiteside et al. [50] prepared multilayer h-BN structures perpendicular (Figure 7a) to the substrate on AlGaN/GaN (Figure 7b) and compared the two-dimensional electron gas (2DEG) values before sputtering, after sputtering, and after annealing. It was found that the increased h-BN did not affect the electronic properties of the device. It was proved that h-BN can work as a passivation layer for efficient heat dissipation without affecting the performance of the device (Figure 7c) [50].

**Figure 8:**
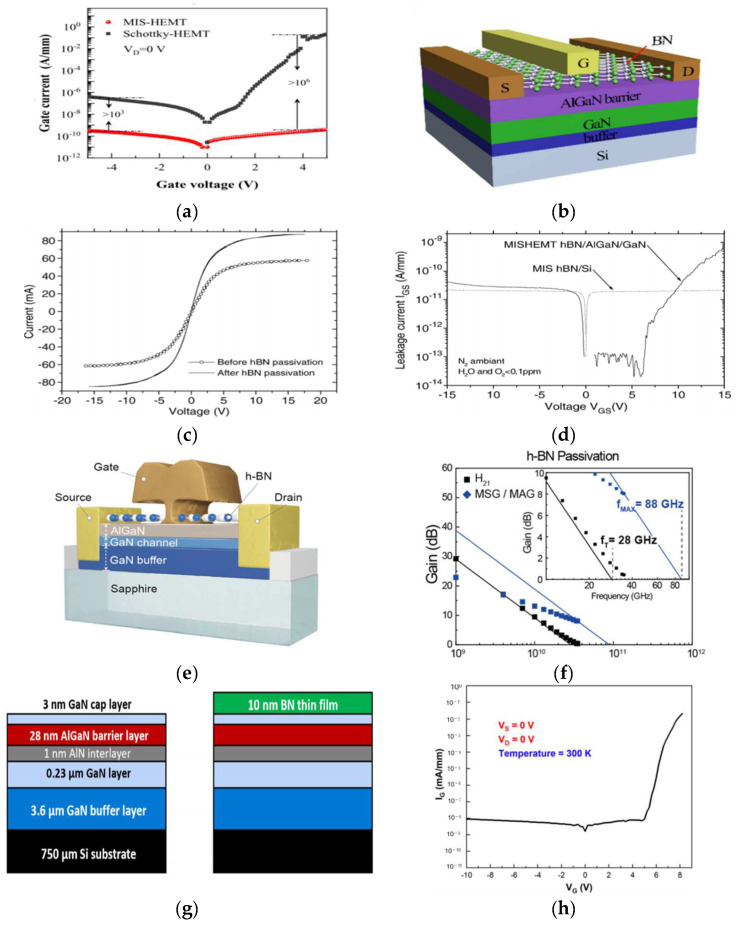
(**a**) Schematic structure of MIS-HEMT with BN dielectric. (**b**) The gate leakages current as a function of gate voltage, while the drain voltages were kept at 0 V [51]. (**c**) I–V measurement between two ohmic contacts with a spacing of 5 µm in an AlGaN/GaN structure, before and after passivation with a thick 25 nm hBN thin film. (**d**) I_GS_–V_GS_ characteristic of Au/Ni/h-BN (25 nm)/AlGaN/GaN/Al_2_O_3_ MIS-HEMT structure with LG = 2 µm and W = 100 µm [52]. (**e**) Schematic cross-section of the fabricated AlGaN/GaN HEMT. (**f**) Transfer characteristics, drain current, and transconductance as a function of gate bias [53]. (**g**) The structure of the BN/AlGaN/GaN HEMTs. (**h**) I_G_–V_G_ curve of the ECR-MPCVD-BN/AlGaN/GaN MIS-HEMTs [54].

It is well known that the Schottky gates in AlGaN/GaN high electron mobility transistors (HEMTs) exhibit high leakage current and that oxygen and nitrogen vacancies are present on the AlGaN surface. These are the major factors limiting the performance and reliability of GaN HEMTs for high-power microwave frequency applications. Bing Ren et al. [51] prepared a novel α-BN/h-BN dual-layered dielectric to fabricate an AlGaN/GaN metal–insulator–semiconductor high-electron-mobility transistor (MIS-HEMT). The electrical properties of the MIS-HEMT were characterized in comparison to the conventional Schottky-HEMT, as shown in Figure 8d. The gate leakage current (drain voltage = 0 V) is shown in Figure 8e. Compared with the Schottky-HEMT, the reverse and forward gate leakage currents of the MIS-HEMT were significantly suppressed by more than three orders and six orders of magnitude, respectively. Gerbedoen et al. [52] prepared an AlGaN/h-BN/GaN heterostructure, which resulted in a significant reduction of the gate leakage current. The photoluminescence surfacetate spectroscopy (PLS^3^) technique was used to evaluate the surface state density on the h-BN/AlGaN interface, presenting a minimum value around 5 × 10^11^ cm^−2^ eV^−1^ at −1.46 eV under the conduction band. The fabricated MIS-HEMTs showed a low leakage current—lower than 20 pA/mm at +16 V. This proved that h-BN films can be used as a passivation layer and a gate dielectric on AlGaN to neutralize surface traps. Seokho Moon et al. [53] prepared a AlGaN/GaN high-electron-mobility transistor with an h-BN passivation layer. The fabricated AlGaN/GaN HEMT with h-BN showed a very promising performance, including a cutoff frequency (f_T_) and maximum oscillation frequency (f_MAX_) as high as 28 and 88 GHz, respectively, enabled by an effective passivation of surface defects on the HEMT wafer to deliver accurate information with minimized power loss. Tsung-Han Yang et al. [54] prepared the AlGaN/GaN MIS-HEMTs with electron cyclotron-resonance microwave plasma chemical-vapor-deposition (ECR-MPCVD), depositing BN as a gate dielectric. The h-BN could effectively reduce the leakage current and improve device performance.

### 2.3. h-BN/III–V LED

**Figure 9:**
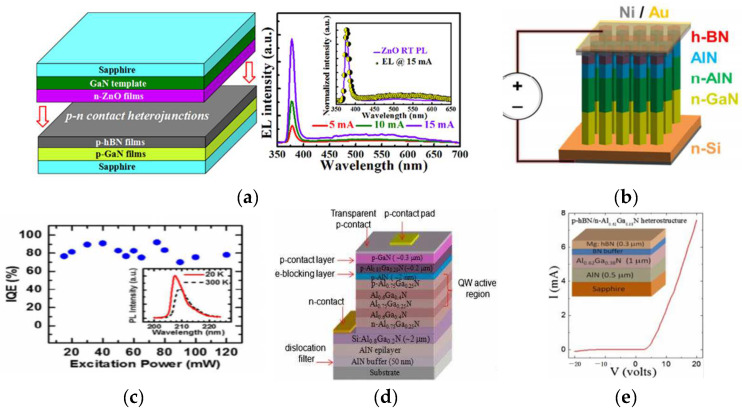
(**a**) High-performance ultraviolet light-emitting diodes were achieved by using n-ZnO/p-hBN/p-GaN contact heterojunctions [56]. (**b**) Schematic of the fabricated LED structure. (**c**) Estimated IQE at these excitation powers [57]. (**d**) Schematic layer structure. (**e**) I–V characteristics and schematic illustration of a p-BN:Mg/n-Al0.62Ga0.38N/AlN p–n structure in which the buffer layer was doped with Mg and p-contacts were annealed at 1020 °C exhibiting a diode behavior [33].

UV-LEDs have been paid more attention due to their important applications. GaN is the most commonly used material for UV-LEDs. ZnO has a wide direct band gap (3.37 eV) and a high exciton binding energy (60 meV), which makes it a highly efficient UV light-emitting semiconductor material with much higher luminescence efficiency than GaN. Due to the difficulty in obtaining stable and repeatable p-type ZnO thin films, there is still a weak electroluminescence (EL) phenomenon in ZnO-base homogeneous LEDs. To overcome this problem, the n-ZnO/p-GaN structure was prepared in [55]. Because of the lattice mismatch between the materials, the quantum efficiency decreased and the relatively high emission open current density made the luminescence efficiency lower. As a material with a wide band gap and low electron affinity, h-BN has potential in UV-LEDs. Gaoqiang Deng et al. [56] used Mg-doped h-BN to prepare n-ZnO/p-hBN/p-GaN (Figure 9a). The use of h-BN greatly improved the luminescence efficiency and reduced the adverse effects caused by lattice mismatch. AlGaN is an ideal material for deep UV optoelectronic devices, but its luminescence efficiency is low in a specific band. David et al. [57] achieved excellent luminescence performance (up to 80% at 20 A/cm^2^) from an Al(Ga)N/h-BN light-emitting LED (Figure 9b,c). H.X. Jiang et al. [33] prepared a novel DUV emitter layer structure based on a hexagonal boron nitride (h-BN) and AlGaN p–n junction (Figure 9d). The p-hBN/n-AlGaN heterostructures revealed decent diode behaviors with very low reverse leakage currents (Figure 9e).

**Figure 10:**
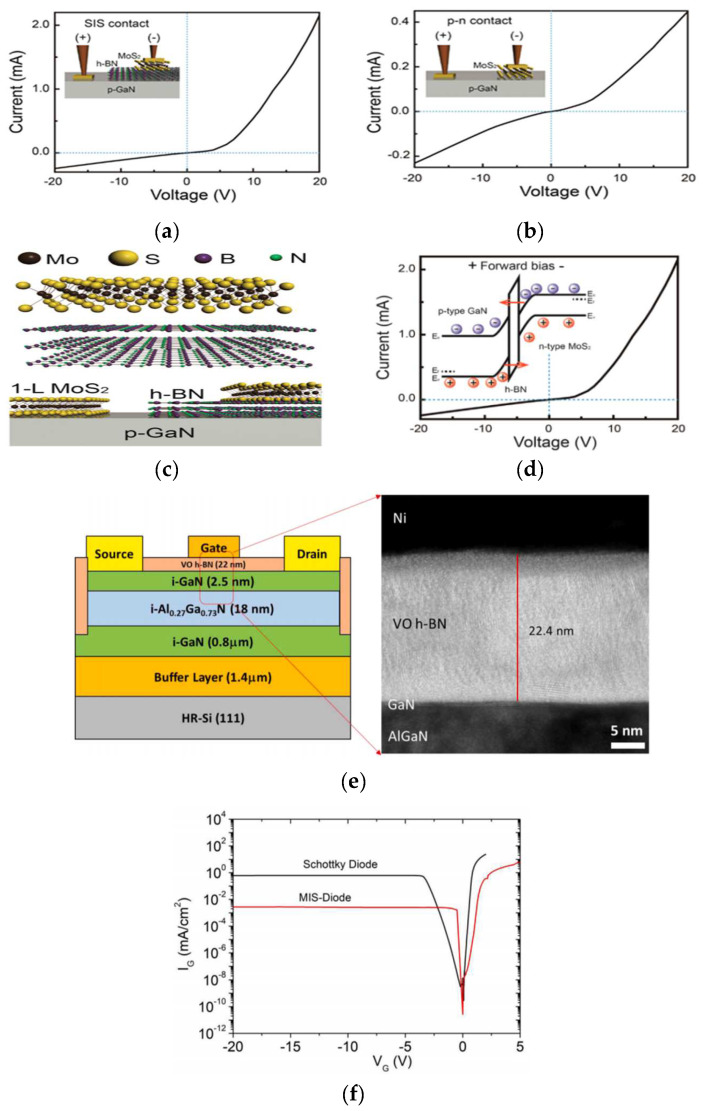
I–V curves of the (**a**) SIS and (**b**) p–n heterojunction structures. A higher threshold voltage with a much higher current was observed for the SIS structure compared to that of the p–n structure. (**c**) Cross-sectional schematics of the SIS and p–n heterojunction structures. (**d**) Energy band diagrams of the SIS heterojunction structure [65]. (**e**) Schematic cross-sectional diagram of VO h-BN AlGaN/GaN MIS-HEMT on a silicon substrate with a corresponding TEM scan under the gate. (**f**) Two terminal gate leakage current characteristics of a conventional Schottky diode and a VO h-BN AlGaN/GaN MIS-diode as a function of voltage [66].

In recent years, 2D transition-metal dichalcogenides (TMDs) with high transparency and thin atomic layer structures [58,59] have been widely used in nanoelectronics and optoelectronics [60,61,62]. Among typical diodes, semiconductor–insulator–semiconductor (SIS) diodes have considerable advantages over traditional p–n junction diodes. These advantages include the lack of a depletion region and a high current flow as a result of carrier tunneling [63,64]. Hyun Jeong et al. [65] prepared MoS_2_/GaN and MoS_2_/h-BN/GaN structures. Clear current rectification characteristics were observed in both structures (the p–n and SIS heterojunction diode). In particular, the threshold voltage of the SIS structure was higher than that of the p–n structure. This showed that h-BN has a broad prospect in the preparation of high-performance diodes. Matthew Whiteside et al. [66] prepared vertically-ordered (VO) h-BN/AlGaN/GaN metal–insulator–semiconductor high-electron-mobility transistors on a Si substrate. In comparison to a conventional Schottky diode, the VO h-BN diode exhibited about two orders of magnitude lower gate leakage current at −20 V and two orders of magnitude lower forward current at +2 V (Figure 10f).

## 3. The Transfer and Preparation of h-BN

### 3.1. The Transfer of h-BN

Due to the difficulty of growing h-BN directly on III–V systems, high-quality h-BN films are currently grown on metal and sapphire substrates and then transferred to III–V systems. At present, the existing transfer methods can be divided into wet transfer and dry transfer.

**Figure 11:**
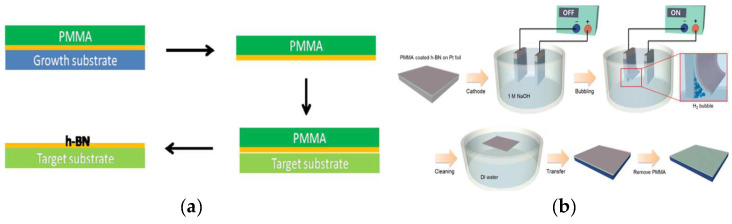
(**a**) PMMA transfer [67]. (**b**) Electrochemical bubbling-based method used to transfer the h-BN layer [68].

Wet transfer includes the PMMA method (Figure 11a) and the bubble method (Figure 11b). A PMMA layer is coated on the h-BN film, the substrate is etched after drying, and the PMMA layer is removed with acetone. Then, the obtained h-BN film is transferred to the III–V system. This method can transfer h-BN films on a large scale, but the residual PMMA will affect the quality of the device. The bubble method is an improvement on the PMMA method. The substrate coated with PMMA layer is placed in an electrolytic pool, and current is passed through. The vapor generated on the metal surface is used to separate the substrate and h-BN film, and then, the PMMA layer is removed with acetone. The bubble method does not etch the metal substrate and reduces the cost. It can realize the recycling of the metal substrate.

**Figure 12:**
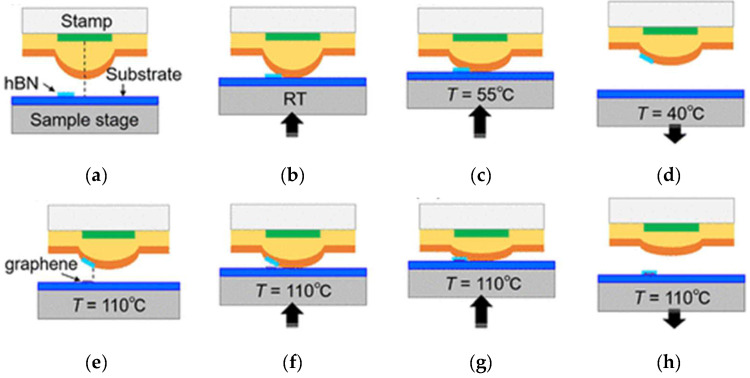
Schematic illustration of the transfer process flow [69]. (**a**) h-BN flake on a SiO_2_/Si substrate; (**b**) center ofthe stamp begins to contact with the substrate; (**c**) circular contact area covers the h-BN flake. (**d**) picked up h-BN flake on the stamp surface (**e**) graphene flake on the substrate; (**f**) stamp having the h-BN flake begins to contact with the substrate having the graphene flake; (**g**) circular contact area covers the graphene flake, so that the h-BN flake is overlapped with the graphene flake; (**h**) h-BN/graphene stack.

Dry transfer uses no PMMA; instead, it uses a technique with a viscoelastic polymer stamp. First, the h-BN film is glued on the substrate. The substrate then heats up and moves up with the platform, transferring the h-BN film onto the graphene (Figure 12a–h). This method has no PMMA residue and avoids ruptures in the film caused by bubbles so that the prepared h-BN film can be completely transferred.

### 3.2. Chemical Vapor Deposition (CVD)

**Figure 13:**
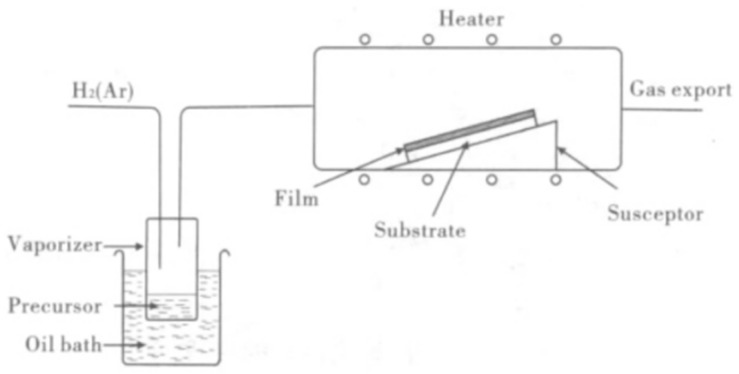
Schematic diagram of the MOCVD principle [70].

CVD is a technique in which a mixture of precursors interacts with the substrate surface at a relatively high temperature to decompose some components in the mixed gas and then forms a solid film, a coating of metal, or a compound on the substrate surface (Figure 13). The basic principle of CVD technology includes the reaction principle and the thermodynamic principle, and reaction types include the thermal decomposition reaction, the redox reaction deposition, the chemical synthesis reaction deposition, the chemical transport reaction deposition, and so on. The reaction generally occurs in five stages: (1) the reactants pass through the boundary layer; (2) the reactants adsorb onto the substrate surface; (3) a chemical deposition reaction; (4) some products diffuse to the boundary layer; and (5) the products and reactants enter the main airflow and leave the system. CVD can be divided into metal–organic chemical vapor deposition (MOCVD), low pressure chemical vapor deposition (LPCVD), APCVD, etc. The choice of substrates, precursors, and various growth conditions affect CVD growth, which will be discussed in detail in the following text.

#### 3.2.1. The Choice of Substrates for CVD Growth

In recent years, metal substrates have been the focus of research for the preparation of highly crystalline h-BN films with CVD. Metal substrates have both substrate and catalytic functions. Compared to sapphire, metal substrates are easier to process and have a better surface morphology. This section summarizes three commonly used metal substrates: copper, platinum, and nickel.

##### Cu Foil

**Figure 14:**
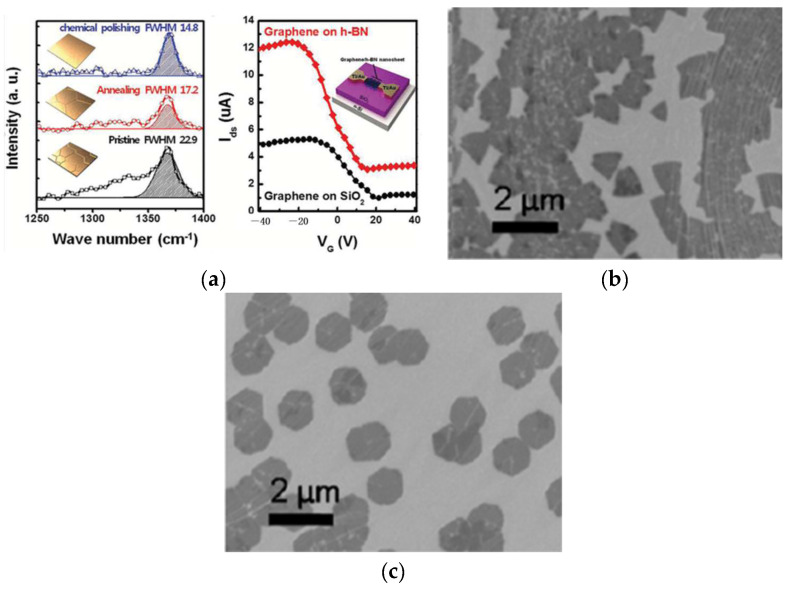
(**a**) Raman spectra of h-BN nanosheets grown on individual Cu foils at 514 nm. The inset images reveal the schematic illustrations of the morphology-controlled Cu foils and the I_ds_–V_g_ characteristics of graphene (GR) TFTs on bare SiO_2_ and high-quality h-BN nanosheets in atmospheric ambience [71]. (**b**,**c**) SEM images of (**a**) triangular and (**b**) hexagonal-shape h-BN domains grown on unpolished and polished Cu, respectively [72].

The smoothness of the surface of a substrate is a prerequisite for the growth of highly crystalline films. The surface morphology of the substrate not only affects the quality of films but also changes the shape of the crystal. Lee et al. [71] conducted APCVD growth of h-BN on copper foils of various surface morphologies. The number of impurity particles in the h-BN nanosheets grown on the polished Cu foil was decreased as compared to that of the h-BN nanosheets grown on pristine Cu foil. The performance of graphene devices deposited on high-quality h-BN film was increased by two times compared to a SiO_2_ substrate (Figure 14a). Roland et al. [72] conducted the CVD growth of h-BN on copper foil. The films grown on unpolished substrates were triangular, and those grown on polished substrates were hexagonal (Figure 14b,c). The grain size of the latter was much larger than that of the former, and the film was smoother. It was easier to prepare single crystals of large-size graphene on Cu substrates than on h-BN. This may be due to the stronger chemical affinity of intermediates containing N to Cu surfaces than intermediates containing C [73]. Therefore, a series of substrate treatments are required to improve the grain density when designing an experimental scheme.

**Figure 15:**
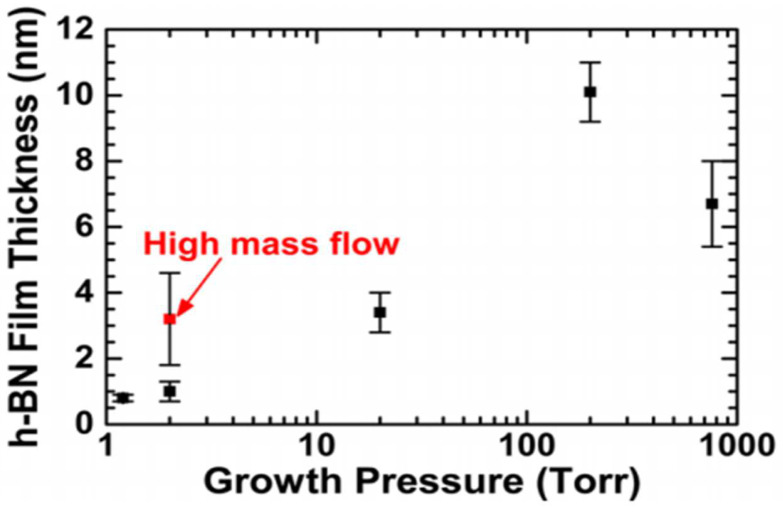
h-BN film thickness versus growth pressure, showing thin h-BN films at LPCVD [74].

The key to the preparation of high-performance electronic devices lies in the good control of crystal quality and the number of layers. Pressure is an important factor affecting film thickness in CVD. Koepke et al. [74] synthesized h-BN film on Cu substrate with CVD under different pressures. The experiment showed that thin and smooth films were synthesized when the pressure was very low (Figure 15), while thick films were synthesized under medium and high pressure with poor purity and low crystallinity. This phenomenon is attributed to the incomplete decomposition of precursor due to Cu substrate surface passivation under high pressure, a low growth rate of film, and the introduction of impurities.

**Figure 16:**
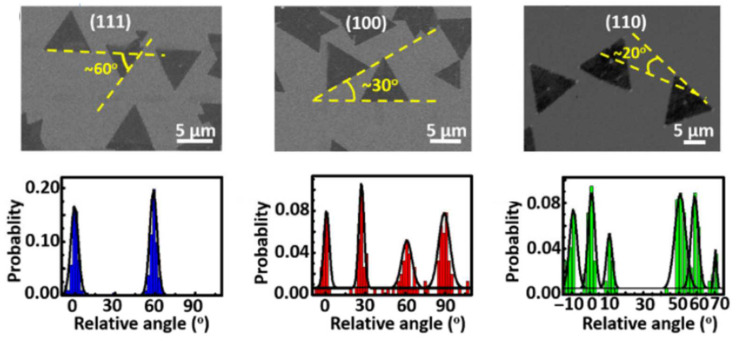
Representative SEM images of h-BN grown on Cu (111), Cu (100), and Cu (110). Statistical distributions of the edge angles of individual triangular h-BN domains grown on Cu (111), Cu (100), and Cu (110) faces [75].

Cu has three main orientations: Cu (111), Cu (100), and Cu (111). Song et al. [75] studied the growth conditions of thin films with different substrate orientations and found that the growth conditions on different orientations were different. Among them, a triangle h-BN with a uniform shape was grown on a Cu (111) surface, and Cu (111) was found to be the best orientation for growth (Figure 16).

**Figure 17:**
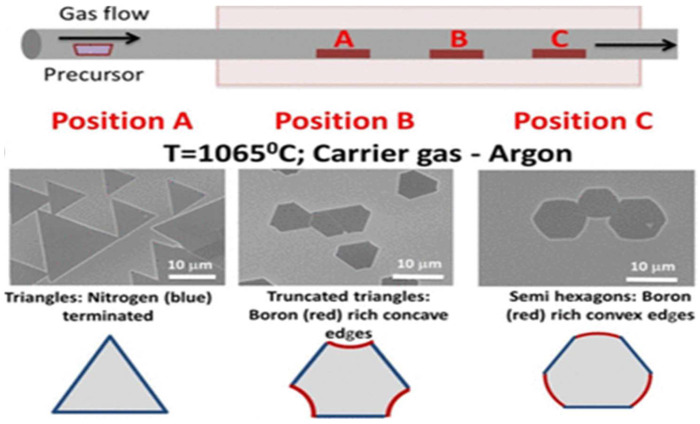
APCVD experimental setup for h-BN growth and APCVD experimental setup for h-BN growth [76].

The position of the substrate in the reaction chamber also affects film growth. Yijing Stehle et al. [76] placed three Cu substrates at different positions in the CVD reaction chamber, and the h-BN crystals grown on the three substrates showed different shapes (Figure 17). In the position away from the air inlet, the h-BN crystal gradually changed from a triangle to a hexagon. The reason for this phenomenon is that the N/B at each position is not uniform. Different N/B proportion influence the growth rate in all directions and ultimately lead to different crystal shapes.

##### Pt Substrate

**Figure 18:**
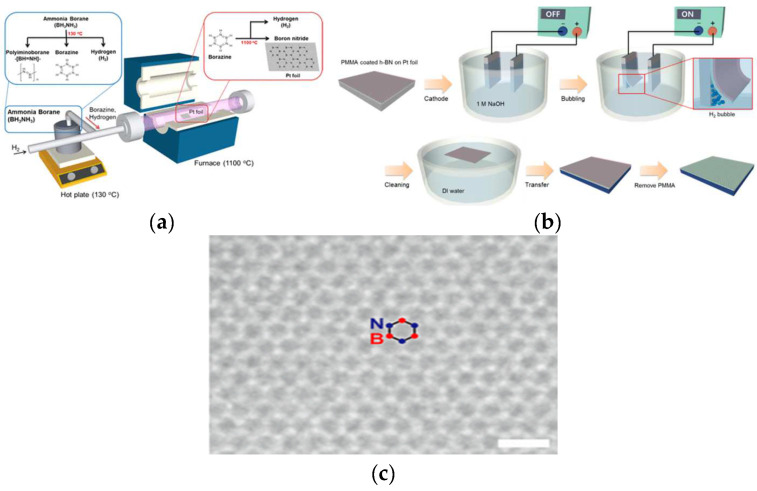
(**a**,**b**) Schematic diagrams of the LPCVD system used for h-BN growth and the electrochemical bubbling-based method used to transfer the h-BN layer. (**c**) Atomic-resolution TEM image of single-layer h-BN [68].

Gwangwoo et al. [68] used the LPCVD method to synthesize the high-quality monolayer h-BN on a Pt foil (Figure 18a). h-BN thin films grown on Pt foils were transferred to SiO_2_/Si substrates using electrochemical bubbling (Figure 18b). Pt foils can be reused and can still grow high-quality h-BN films even after 100 growth cycles. More importantly, HRTEM results revealed that the h-BN was a single atom thick with a high degree of crystallinity. Gao et al. [77] reported large-area preparation of h-BN thin films on reusable Pt substrates by using the LPCVD method. The growth of h-BN on Pt is very sensitive to pressure and the morphology of the substrate surface (Figure 18c). The grain size and density also affect growth.

##### Ni Foil

Jing Kong et al. [78] studied the large-area deposition of h-BN thin films on Ni foils. The substrate orientation was found to have a great influence on the deposition. Harry Chou et al. [79] studied the influence of the Ni orientations on deposition: the deposition rate and thickness on the (110) substrate were better than those on the (100) and (111) orientation substrates. The experimental results showed that Ni had a catalytic effect on the growth of h-BN thin films, which is reflected in the increased growth rate. However, the mechanism of film growth and the reason for metal catalysis are still unclear.

##### Nonmetallic Substrates

**Figure 19:**
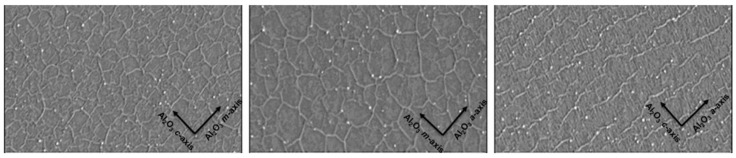
AFM images of h-BN grown on a-plane, c-plane, and m-plane sapphire [82].

Nonmetallic substrates mainly include sapphire, Si/SiO_2_, GaN, AlN, etc. Compared to metal foils, nonmetal substrates are more widely used and easier to obtain but are inferior to metal substrates in terms of film quality (Table 4). Hokyeong Jeong et al. [80] grew high-quality h-BN thin films on a Ni (111) foil by using MOCVD. Compared to inert sapphire substrates, the catalytic Ni (111) template proved favorable for the rapid growth of high-quality h-BN films at relatively low temperatures (1000 °C). In addition, other temperature-related effects, such as surface reaction and etching, may seriously limit the controllable realization of smooth h-BN monolayers [73]. The use of a buffer layer can improve the crystal quality of BN film. Smooth and high-quality h-BN films have been reported on a sapphire substrate that contained an AlO_x_N_y_ buffer layer [81]. Phuong Vuong et al. [82] prepared an h-BN film on the different kinds of sapphire. The root-mean-square (rms) roughness values of h-BN grown on a-, c-, and m-plane sapphire substrates were 3.2, 3.7, and 2.7 nm, respectively (Figure 19).

#### 3.2.2. Precursors

The composition, structure, and chemical properties of precursors (Table 5) in CVD growth have a strong influence on the selection of parameters and the quality of products. At present, organic compounds, including B(C_2_H_5_)_3_ [78], B(OCH_3_)_3_ [86], borazine, and boranes (including eboranes and maleboranes) are being used for controllable growth. Kim et al. [87] used borazine and hydrogen at 1100 °C for the controllable and repeatable growth of multilayer h-BN thin films on iron foils. Adams et al. [88] synthesized BN thin films by using ethyl borane as a nitrogen source. Nakamura et al. [89] prepared BN thin films by using B(C_2_H_5_)_3_ as a boron source. Singhal Ranjan et al. [90] deposited h-BN thin films using borane ammonia and H_2_ on the sapphire substrate. The reactions in organic boron sources include decomposition, reaction synthesis, and re-decomposition. h-BN growth using organic boron sources has very strict requirements on the growth temperature and pressure, and the films grown under appropriate conditions have high crystallinity and good quality. One of the major limitations of organic boron sources is their high cost. The material source is difficult to preserve and toxic, and the products can easily carry impurity elements, such as H, C, and O, from the sources. The next step is to explore the intermediate processes of the reaction, including the intermediates and byproducts of the reaction, the removal of impurities, and the most appropriate temperature and pressure for the reaction.

Currently, the mainstream halide precursors for h-BN growth include BCl_3_ [96], BF_3_ [92], BBr_3_ [97], etc. Osamu et al. [98] prepared high-crystallinity h-BN films with BCl_3_-NH_3_ as the precursor. T. Sugino et al. [99] prepared h-BN films with BCl_3_-N_2_ as the precursor system. The advantages of boron halide sources are the low price of raw material, easy industrialization, simple reaction process, fewer byproducts (mainly tail gas), simple reaction equipment, and low cost. The disadvantages are the low crystallinity of the grown film and corrosive exhaust generated by the reaction that corrodes the film, substrate, and instrument.

#### 3.2.3. Reaction Conditions

In addition to the substrate and precursor, the reaction temperature, pressure and B/N element ratio greatly affect the reaction speed. When the ratio of N/B elements is large, the growth rate of h-BN is stable and slow at a moderate temperature, but the film has high crystallinity. When the ratio of N/B elements is small, the growth rate is significantly accelerated, resulting in more defects in the film and low crystallinity [73]. At this point, the temperature and pressure slow down the growth rate effectively. In the design of an experimental scheme, the reaction conditions cannot be ignored. It is very important to balance the three to prepare high-quality thin films.

### 3.3. Physical Vapor-Deposition (PVD)

**Figure 20:**
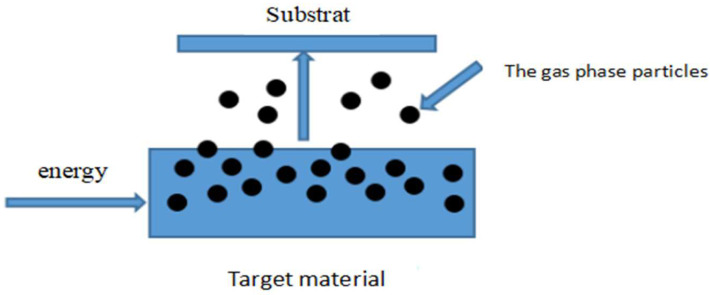
Schematic diagram of PVD.

In the physical vapor-deposition method, the surface of the material source is vaporized into atoms, molecules, and ions in a vacuum, which form a thin film on the substrate surface (Figure 20). The basic steps of PVD growth include: (1) the generation of particles from the material source (evaporation, sublimation); (2) transportation of the particles to the substrate; and (3) film growth from deposition of the particles on the substrate. PVD can be divided into vacuum evaporation, sputtering, and sediment. At present, electron beam evaporation and magnetron sputtering are widely used and mature PVD methods.

#### 3.3.1. E-Beam

Electron-beam evaporation is an important method of coating. Its principle is relatively simple: the material is heated by an electron beam in a vacuum, and when a certain temperature is reached, the material evaporates and is finally deposited on the substrate. Nasrazadan et al. [100] obtained low-quality BN thin films by using electron-beam evaporation. N_2_ gas can be partially formed during the B/N vaporization and transport process. In addition, the electron beam hits the material target at a very high temperature, and the high temperature decreases the adhesion coefficient between the N atoms and the substrate. If not enough N_2_ is not supplemented in the preparation process, the N content is eventually lost in the film. The direct evaporation of h-BN powder results in fast deposition, making it difficult to form a uniform and stable few-layer, single-crystal film. To solve the problem of low nitrogen content, Jingze Tian et al. [101] used the plasma immersion ion implantation (PBII) method to prepare the h-BN structure: First, the e-beam evaporation method was used to prepare B film on the substrate, and then a certain dose of N particles was injected. The experiment showed that the quality of the h-BN film increased significantly with an increase in the N dose.

#### 3.3.2. Sputtering

**Figure 21:**
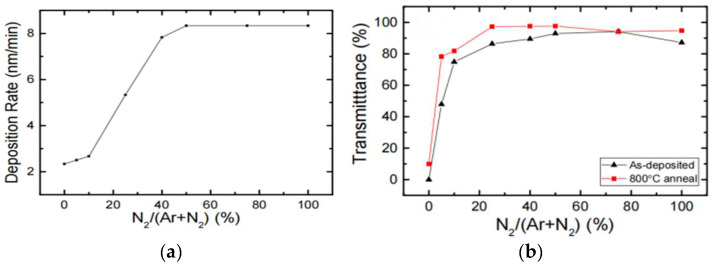
(**a**) Deposition rate as a function of N_2_/(N_2_ + Ar) gas flow ratio. (**b**) Optical transmittance at 265 nm for hexagonal boron nitride (h-BN) films and various N_2_/(N_2_ + Ar) flow ratios before and after post-annealing treatments at 800 °C [104].

Magnetron sputtering technology is a high-speed and low-temperature sputtering technology developed in the late 1970s. Sputtering occurs when high-speed ions hit a solid surface and eject atoms or molecules from the surface through an energy exchange with atoms or molecules. h-BN target is usually used to deposit thin films on sapphire or Si with magnetron sputtering. This is a rapid growth method that results in a polycrystalline or heterogeneous film. Sutter et al. [102] prepared large-area and few-layer h-BN films by using RF sputtering. They used pure B target and Ar/N_2_ to avoid nonuniform crystallization caused by the direct sputtering of cluster molecules so that B and N species could orderly cluster on the surface to form h-BN. With magnetron sputtering, doped h-BN films can be deposited on a GaN surface conveniently. Nose et al. [103] inserted Zn rods during RF sputtering to obtain h-BN films with different doping concentrations. This method was recently used by Deng et al. [56] to grow Mg-doped p-type h-BN that was directly deposited on p-type GaN to realize an h-BN-GaN heterojunction. The fraction of the gas mixture (Ar/N_2_) also has a great influence on the growth of h-BN in the sputtering process. GuoDong Hao et al. [104] demonstrated the deposition of a thick h-BN film by using low-temperature RF sputtering on an Al_0.7_Ga_0.3_N layer and compared the effects of different gas ratios on the growth of h-BN thin films. The experimental results showed that the deposition rate and optical transmittance of the film are improved with an increase in the fraction of the gas mixture (Ar/N_2_) (Figure 21a,b).

### 3.4. Co-Segregation Method

In alloys, the redistribution of atoms between the interface and the body leads to the phenomenon of solute solubility at the interface, in which the concentration at the interface is much greater than in the body, called segregation. In the co-segregation method, this phenomenon is used to prepare h-BN films.

**Figure 22:**
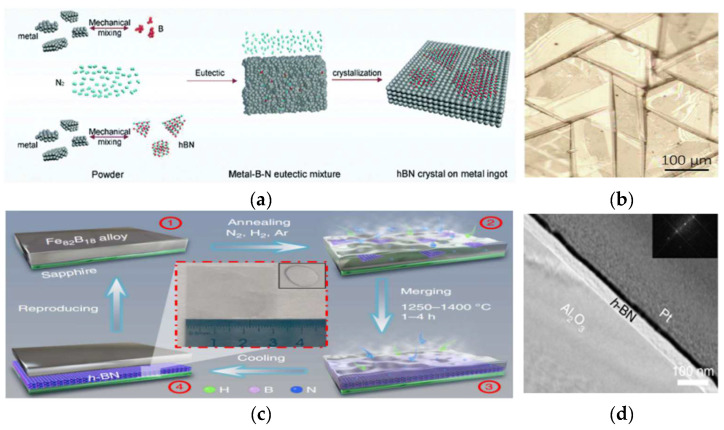
(**a**) Schematic of the procedure of h-BN growth. (**b**) Zoom-in optical image of h-BN films showing grain boundaries that roughly follow three directions. (**c**) Schematics of multilayer h-BN grown on sapphire with Fe_82_B_18_ alloy and nitrogen as reactants. (**d**) Corresponding TEM images of multilayer h-BN with different thicknesses [105,106].

The co-segregation method requires metallic materials that can be used as B and N solvents. The two elements can be precipitated on the surface and combined into h-BN by controlling the temperature. Iron is used as one of the solvents. Li Yifei et al. [105] prepared an h-BN thin film using Fe as a cosolvent. Figure 22a shows the growth scheme. B and Fe powder were mixed in an N_2_ atmosphere and heated to a certain temperature to generate a thin film on the iron surface. Zhiyuan Shi et al. [106] prepared an h-BN film with controllable thickness by using the vapor–liquid–solid growth method. In this method, they heated Fe_82_B_18_ alloy on the sapphire substrate to a molten state and then passed a mixture of N_2_, H_2_, and Ar gases, obtaining an alloy/h-BN/substrate heterostructure upon cooling. After exfoliation of h-BN from the alloy surface, the alloy can be recycled for secondary utilization, as shown in Figure 22c.

**Figure 23:**
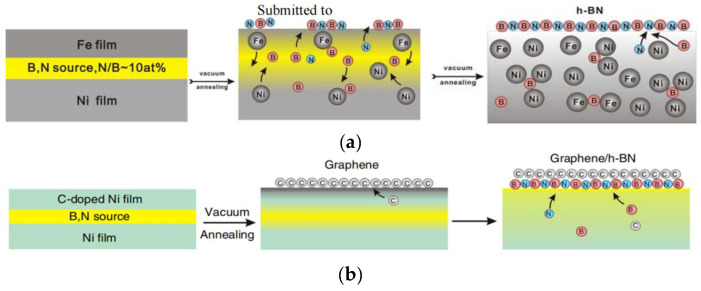
Hexagonal boron nitride thin films were prepared with the co-segregation method [67]. (**a**) the system of Fe-B/N-Ni; (**b**) the system of Ni-B/N-Ni.

Another solvent is nickel: Zhang Zhaohua et al. [67] systematically studied the use of nickel as a solvent: (1) Electron beam-evaporated high-purity boron nitride was used as a source of B and N, where Ni was used as a substrate. A layer of Fe film on top was used to absorb impurities. The multilayer structure was then annealed in an N_2_ atmosphere. B and N diffused to the surface to form a hexagonal phase structure. Due to Fe’s strong ability to capture C and O at high temperatures, the resulting film had fewer impurities, as shown in Figure 23a. (2) With Ni as the substrate, electron beam-evaporated high-purity BN was used as the source of B and N, on which a layer of C containing Ni was deposited. The multilayer structure was then annealed in an N_2_ atmosphere. B and N diffused to the surface to form a hexagonal phase structure, and C diffused to the outermost layer to form a graphene structure. The graphene/h-BN heterostructure was prepared, as shown in Figure 23b.

Large-area h-BN films can be synthesized with the co-segregation method, but due to the poor crystallinity of the films, they cannot be used as materials for high-precision electronic devices. After precipitation on the substrate surface, the prepared film needs to be exfoliated and transferred using other methods that are not suitable for direct deposition on the surface, such as GaN, which affects the degree of bonding between the two heterogeneous structures.

## 4. Summary and Outlook

h-BN and other van der Waals crystals have been the focus of research for their great potential in various device applications. At present, there are fewer studies on the h-BN/III–V structure system, which has broad prospects for use in UV-LEDs, photoelectric detection, and transistors. In the case of GaN-based light-emitting devices, BN provides efficient electron blocking and hole injection into the active region. For transistor structures, h-BN acts as a passivation layer and gate dielectric where h-BN neutralizes surface traps and effectively reduces leakage current. In photodetectors, it reduces the dark current. So far, CVD is the main source of high-quality h-BN thin films, which can prepare device-grade h-BN films with a controlled number of layers. However, the prepared h-BN thin films still need to be transferred from the substrate to the device surface through other means. The heterogeneous binding of the film on the device surface is still a great challenge. The growth mechanism of h-BN and the catalytic effect of metal substrates still need to be explored to improve the quality and integration of thin films.

## Figures and Tables

**Table 1:** Main isomer parameters of BN.

	c-BN	h-BN	r-BN	w-BN
Hybrid approach	Sp^3^	Sp^3^	Sp^2^	Sp^2^
Crystal system	Cubic	Hexagonal	Tripartite	Hexagonal
Lattice constant (nm)	a = 0.362	a = 0.251 c = 0.666	a = 0.255 c = 0.421	a = 0.250 c = 0.999
Density(g/cm^3^)	2.28	3.45	3.48	2.28

**Table 2:** Comparison of h-BN and graphite properties [14,24,28].

	h-BN	Graphite
Lattice constant (nm)	a = 0.2504 c = 0.6661	a = 0.2456 c = 0.6696
Density (g/cm^3^)	2.0–2.2	2.3
Electrical conductivity	Insulators	Conductor
Thermal conductivity	High	High
Band gap (eV)	5.9	0

**Table 3:** Comparison of h-BN with GaN and AlN [33].

	h-BN	GaN	AlN
Lattice constant (nm)	a = 0.2504 c = 0.6661	a = 0.3112 c = 0.4982	a = 0.3186 c = 0.5186
Band gap (eV)	5.9	6.1	3.4
Band gap type	Direct band gap	Direct band gap	Direct band gap
Thermal conductivity (W/m·K)	600	285	130
Hole concentration (cm^−3^)	10^18^–2.7 × 10^19^	10^12^	10^17^–10^18^
Electron concentration (cm^−3^)	2 × 10^19^	7.3 × 10^14^	3 × 10^19^
Electron mobility (cm^2^/V·s)	48	426	440
Hole mobility (cm^2^/V·s)	2–26	<5	10

**Table 4:** A summary of the growth of 2D h-BN synthesized on various substrates using CVD.

Substrates	Precursor	Key Parameter	Layer	Ref.
Cu foil	Ammonia borane	Cu morphology	6–8 layers	[71]
Ni film	Borazine	Precursor dosage	5–50 nm	[78]
Pt foil	Ammonia borane	Repeated growth	Monolayer	[68]
SiO_2_	Ammonia borane	Growth time	Few-layers	[83]
Sapphire	Ammonia borane	Epitaxial growth	Few-layers	[84]
AlN	TEB	Temperature	Monolayer	[85]

**Table 5:** This table summarizes the precursors, growth conditions, and quality of CVD-grown h-BN.

Precursors	Quality	Growth Conditions	Ref.
Borazine and H_2_	Large-area, multilayer h-BN film with strong cathodoluminescence and high mechanical strength	CVD growth at 1100 °C for 30 min on Fe foil	[87]
BCl_3_, NH_3_, N_2_, and H_2_	The maximum thickness was about 10 nm	APCVD growth at 1000 °C	[91]
BF_3_ and NH_3_	The h-BN was obtained when no self-biss was applied to the substrate	PECVD growth in a temperature range of 583–793 k	[92]
Ammoina borane	Single and multilayers were grown on various substrates	APCVD and LPCVD growth in a temperature range of 700–1100 °C	[71,93,94,95]

## Data Availability

The data presented in this study are available upon request from the corresponding authors.
